# Deacetylation by SIRT1 promotes the tumor-suppressive activity of HINT1 by enhancing its binding capacity for β-catenin or MITF in colon cancer and melanoma cells

**DOI:** 10.1038/s12276-020-0465-2

**Published:** 2020-07-07

**Authors:** Taek-Yeol Jung, Gyu-Rin Jin, Young-Bin Koo, Mi-Mi Jang, Chan-Woo Kim, Soh-Yeon Lee, Hyelee Kim, Chae-Young Lee, Soo-Young Lee, Bong-Gun Ju, Hyun-Seok Kim

**Affiliations:** 1 Department of Life Science, College of Natural Science, Ewha Womans University, Seoul, 03760 South Korea; 2 Department of Life Science, College of Natural Science, Sogang University, Seoul, 04107 South Korea; 3 Department of Biochemistry, College of Medicine, The Catholic University of Korea, Seoul, 06591 South Korea; 4 The Research Center for Cellular Homeostasis, Ewha Womans University, Seoul, 03760 South Korea; 5 Department of Bioinspired Science, Ewha Womans University, Seoul, 03760 South Korea

**Keywords:** Cell growth, Cancer models

## Abstract

Histidine triad nucleotide-binding protein 1 (HINT1), which belongs to the evolutionarily conserved HIT superfamily, has been shown to possess a tumor-suppressive function by binding to and inhibiting several oncogenic transcription factors, such as β-catenin and microphthalmia transcription factor (MITF), in various types of cancer cells. However, the regulatory mechanism that mediates the binding capacity of HINT1 for partner transcription factors remains elusive. Here, we report that HINT1 is acetylated by CBP at K21 and K30 and deacetylated by SIRT1. Deacetylation of HINT1 by SIRT1 increases the capacity of HINT1 to bind to β-catenin or MITF. As a result, the tumor-suppressive function of HINT1 is increased. In support of this, the deacetylation mimetic HINT1 mutant HINT1 2KR was found to significantly reduce cellular proliferation in colon cancer and melanoma cells and tumorigenesis in xenograft assays. Thus, this study reveals an acetylation-dependent regulatory mechanism that governs the tumor-suppressive function of HINT1.

## Experimental & molecular medicine: tumor suppression: minor modifications aid cancer control

A crucial modification to the tumor-suppressing gene HINT1 helps slow the spread of colon cancer and melanoma according to researchers in South Korea. HINT1 is known to bind to and inhibit several tumor-promoting transcription factors, but it is unclear how this process is regulated. Hyun-Seok Kim at Ewha Womans University in Seoul and co-workers focused on SIRT1, an enzyme that deacetylates, i.e., removes acetyl groups from, various important proteins. They found that the deacetylation of HINT1 by SIRT1 promotes the capacity of HINT1 to bind to transcription factors, thereby enhancing its tumor-suppressing function. Mutant colon cancer and melanoma cell lines with completely deacetylated HINT1 showed significantly reduced growth. The researchers suggest that acetylation and other reversible modifications of HINT1, such as phosphorylation, could be useful in clinical treatments.

## Introduction

Histidine triad nucleotide-binding protein 1 (HINT1) is a member of the evolutionarily highly conserved HIT superfamily, which is divided into three branches, namely, the histidine triad nucleotide-binding protein (Hint) branch, the fragile histidine triad (Fhit) branch, and the galactose-1-phosphate uridyltransferase (GalT) branch. HINT1 acts as a nucleotide hydrolase and/or possesses transferase activities^1^. Previously, many studies have suggested that HINT1 can serve as a novel tumor suppressor in various types of cancer, such as melanoma, gastric cancer, and colon cancer, and may also have an important role in the treatment of neuropsychiatric diseases^2,3^. Preliminary evidence for HINT1’s function in tumorigenesis comes from homozygous or heterozygous deletion of HINT1 in mice, which caused a marked increase in susceptibility to chemical carcinogen-induced malignant tumors, such as gastric tumors, mammary tumors, and ovarian tumors, and increased the occurrence of several types of spontaneous tumors with aging^4,5^, indicating that HINT1 may have a role as a haploinsufficient tumor suppressor in several malignant cancer types. In addition, some studies reported that the transcriptional activity of the *HINT1* gene is silenced by methylation of its promoter in some human non-small-cell lung cancers^6,7^. HINT1 overexpression reduces the growth rate of several cancer cells, such as melanoma, colon cancer, gastric cancer, and lung cancer, or induces apoptosis in SW480 and MCF7 cells^6,8–10^, whereas *Hint1* deficiency enhances the growth and spontaneous immortalization of mouse embryonic fibroblasts^4^, supporting the claim that HINT1 might act as a tumor suppressor in some types of cancer.

Surprisingly, accumulating studies have revealed that HINT1 can inhibit tumorigenesis through regulation of gene transcription, not through its enzymatic activity^2,10^. After the discovery that HINT1 binds to and inhibits the activity of microphthalmia transcription factor (MITF) provided the first evidence for an additional role of HINT1 in transcriptional regulation^11^, several transcription factors, including CDK7, AP1, JNK2, USF2, and β-catenin, which can act as oncogenes in several cancer cell types, were also reported to interact with HINT1 and become inactivated^6,12–14^; this further supports the claim that HINT1 may serve as a tumor suppressor via regulation of gene expression. These studies indicate that the association of HINT1 with partner transcription factors may be a critical mechanism for its tumor suppressor function. Indeed, one study demonstrated that when Ap4A, which is produced in a side reaction by LysRS (Lysyl-tRNA synthetase), binds to HINT1, it interferes with the HINT1–MITF interaction, thereby dissociating the repressor HINT1 from MITF^15^. Furthermore, a recent study showed that HINT1 is subjected to K21 acetylation and Y109 in activated mast cells, together with Ap4A-mediated HINT1 release from MITF^16^, suggesting that posttranslational modifications of HINT1 could be a key mechanism for HINT1’s tumor-suppressive role.

One of the most important posttranslational modifications (PTMs) regulating protein function is lysine acetylation, which is a reversible PTM catalyzed by histone acetyltransferases (HATs), Zn^2+^-dependent histone deacetylases (HDACs), or NAD^+^-dependent deacetylases (sirtuins), leading to changes in protein function in various ways^17,18^. Dysregulation of acetylation has been involved in human diseases such as aging, cancer, inflammation, metabolic diseases, and neurodegenerative diseases^17,18^.

Sirtuins, which are a conserved family of NAD^+^-dependent protein deacetylases and are important in responses to cellular stress and homeostasis in mammals, have received increasing attention due to their diverse roles in many physiological processes, including aging, inflammation, metabolic diseases, and tumorigenesis^19,20^. Among the seven members of the sirtuin family (SIRT1–7), SIRT1 has been the most extensively studied member in various cellular processes^19^. Previously, numerous studies have demonstrated that SIRT1 may serve as either a tumor suppressor or promoter in tumorigenesis, depending on cancer type or context, and this finding remains under debate^20–22^. In colon cancers, observations that SIRT1 can act as a tumor suppressor have been reported in some studies^23,24^. For example, overexpression of SIRT1 in a β-catenin-driven mouse model of colon cancer attenuated tumorigenesis through deacetylation and suppression of β-catenin^23^. In addition, SIRT1 knockdown accelerates tumor xenograft formation by HCT116 cells, whereas SIRT1 overexpression suppresses tumor formation^24^. However, other studies have suggested that SIRT1 can serve as a tumor promoter^25,26^: *APC*^min/+^ mice with SIRT1 deletion only in the intestinal epithelium developed fewer intestinal polyps than did control mice^25^. In sum, although it is clear that SIRT1 is implicated in cancer, its precise role in tumorigenesis is still controversial.

A recent study showed that the HINT1-mediated tumor-suppressive function might be modulated by reversible acetylation^16^. In addition, a previous proteomic study revealed that HINT1 might be deacetylated by SIRT1^27^. Based on these reports, this study sought to determine whether SIRT1 can regulate the tumor-inhibiting activity of HINT1 through deacetylation in colon and melanoma cancer cells. The results showed that HINT1 can be reversibly acetylated at lysine 21 and 30 by CBP and SIRT1 in an in vitro and in vivo cell-based context. SIRT1-mediated deacetylation of HINT1 on lysine 21 and 30 enhances the binding of HINT1 to β-catenin or MITF and reduces their transcriptional activity. Indeed, ectopic expression of the HINT1 2KR (K21/30R) mutant mimicking deacetylation status in colon cancer or melanoma cells significantly suppresses the growth and viability of those cells compared to those of wild-type HINT1. The data from this study provide insight into the role and detailed mechanism, indicating that HINT1 is reversibly modified via acetylation by SIRT1 and CBP in colon and melanoma cancer cells.

## Materials and methods

### Cell culture

Cell lines were purchased from the American Type Culture Collection and were maintained as recommended by the supplier. Cells were cultured at 37 °C in humidified air with 5% CO_2_ in Dulbecco’s modified Eagle’s medium or RPMI medium (Corning Incorporated, Corning, NY, USA) containing 10% fetal bovine serum (Corning Incorporated, Corning, NY, USA) and 1% penicillin/streptomycin (Corning Incorporated, Corning, NY, USA). Cell lines tested negative for mycoplasma.

### Western blot analysis and antibodies

Cells were lysed in RIPA buffer (50 mM Tris, pH 8, 150 mM NaCl, 0.1% sodium dodecyl sulfate [SDS], 0.5% Na-deoxycholate, 1% NP-40) mixed with phosphatase inhibitors and protease inhibitors. Soluble proteins were obtained from the supernatant after centrifugation at 13,000 rpm for 30 min. Protein concentrations were quantified using the Bradford assay. Protein lysate was mixed with sample buffer (0.16 M Tris, pH 6.8, 12.5% β-mercaptoethanol, 12.5% SDS, 25% glycerol, 0.05% bromophenol blue) while boiling for 5 min at 95 °C. Protein samples were separated by 10% or 12% sodium dodecyl sulfate polyacrylamide gel electrophoresis (SDS-PAGE) and transferred to 0.45-μm polyvinylidene fluoride (PVDF) membranes (Millipore, USA). The membranes were incubated with the primary antibodies HA (Santa Cruz, Dallas, TX, USA), CBP (Santa Cruz, Dallas, TX, USA), HINT1 (Santa Cruz, Dallas, TX, USA), SIRT1 (Santa Cruz, Dallas, TX, USA), β-catenin (Santa Cruz, Dallas, TX, USA), α-tubulin (Santa Cruz, Dallas, TX, USA), c-MYC (Santa Cruz, Dallas, TX, USA), cyclin D1 (Santa Cruz, Dallas, TX, USA), MITF (Santa Cruz, Dallas, TX, USA), tyrosinase (Santa Cruz, Dallas, TX, USA), CDK2 (Santa Cruz, Dallas, TX, USA), Flag (Sigma-Aldrich, St. Louis, MO, USA), and Ac-Lys (Abcam, USA) at 4 °C overnight. Then, the membranes were washed three times with phosphate-buffered saline with Tween detergent (PBST), incubated with secondary antibodies for 1 h at room temperature (RT), and washed three times with PBST. The immunoblots were detected with ECL solution (Millipore, USA).

### Immunoprecipitation assay

Cells were lysed in immunoprecipitation (IP) buffer (20 mM HEPES, pH 7.0, 180 mM KCl, 0.2 mM EGTA, 1.5 mM MgCl_2_, 20% glycerol, 1% NP-40), and 1 mg protein lysate was mixed with 50 μg of protein G agarose and primary antibody. The mixture was incubated overnight at 4 °C on a rotator. The precipitated proteins were washed with IP washing buffer (20 mM HEPES, pH 7.0, 180 mM KCl, 0.2 mM EGTA, 1.5 mM MgCl_2_, 20% glycerol, 0.1% NP-40) and mixed with loading buffer for western blot analysis.

### In vitro deacetylation assay

To purify the acetylated form of HINT1 protein for in vitro deacetylation assays, HEK293T cells were cotransfected with Flag-HINT1 and CBP for 48 h and then subjected to immunoprecipitation using agarose conjugated with Flag antibody (Sigma-Aldrich, St. Louis, MO, USA). Immunoprecipitated Ac-Flag-HINT1 was eluted using Flag peptides (Sigma-Aldrich, St. Louis, MO, USA) in accordance with the manufacturer’s instructions. S-Flag-SBP-tagged SIRT1 WT or H363Y recombinant proteins were provided by the Department of Biomedical Science, Graduate School, Kyung Hee University, JE Kim. Eluted Ac-Flag-HINT1 was incubated with recombinant SIRT1 WT or H363Y in deacetylation buffer (40 mM HEPES, pH 7.9, 134 mM KCl, 0.4 mM EDTA, 10 mM MgCl_2_), with or without NAD^+^ (1 mM), at 37 °C for 4 h. Then the HINT1 acetylation levels were determined by western blot analysis.

### TOP/FOP reporter assay

The activity of the β-catenin/TCF signaling pathway was measured by a TOP/FOP flash luciferase reporter assay. TOP flash contains a wild-type TCF binding site, and FOP flash has a mutated TCF binding site that is used to measure β-catenin-dependent TCF transcriptional activity induction. Cells were plated in 24-well plates and cotransfected with TOF/FOP plasmid and Renilla plasmid in HEK293T cells. The cells were harvested after 24 h, and both the firefly and Renilla luciferase activities were measured with the DualGlo^®^ Luciferase Assay System (Promega, Madison, WI, USA).

### Quantitative real-time polymerase chain reaction

Total RNA was extracted from cell lines using TRIzol reagent (Invitrogen, ThermoFisher Scientific, Carlsbad, CA, USA) according to the manufacturer’s instructions, and cDNA was synthesized using PrimeScript RT Master Mix (TaKaRa, Japan) in accordance with the manufacturer’s instructions. The amplification of target genes was performed by quantitative real-time polymerase chain reaction (qRT-PCR). qRT-PCR was performed using the SensiMix SYBR Hi-ROX kit (Bioline, London, UK) with the following sets of oligonucleotide primers: β-actin, 5′-AGAGCTACGAGCTGCCTGAC-3′ (forward) and 5′-AGCACTGTGTTGGCGTACAG-3′ (reverse); cyclin D1, 5′-ACAAACAGATCATCCGCAAACAC-3′ (forward) and 5′-CCTCAGGTTCAGGCCTTGCACTG-3 (reverse); c-MYC, 5′-AATGAAAAGGCCCCCAAGGTAGTTATCC-3′ (forward) and 5′-GTCGTTTCCGCAACAAGTCCTCTTC-3′ (reverse); tyrosinase, 5′-GGCCAGCTTTCAGGCAGAGGT-3′ (forward) and 5′-TGGTGCTTCATGGGCAAAATC-3′ (reverse); CDK2, 5′-CTTAAACCCCAGAATCTCCTCATCAA-3′ (forward), and 5′-AAACAGTGCCCTCCGAGTAATCAT-3′ (reverse). The expression level of the indicated mRNAs was quantified using the 2^−ΔΔCt^ method.

### Chromatin immunoprecipitation (ChIP) assay

Transfected cells were crosslinked for 10 min in 1% formaldehyde. Chromatin was sonicated to obtain fragments of between 200 and 500 base pairs (bp). Crosslinked chromatin was immunoprecipitated with protein G agarose and primary antibody β-catenin (Santa Cruz, Dallas, TX, USA) overnight at 4 °C. Beads were washed with IP washing buffer and TE buffer at 50 nM. The immunoprecipitated chromatin was eluted from protein G agarose by heating at 65 °C and was reverse crosslinked by holding overnight at 65 °C. Eluted DNA was purified using a PCR purification kit (Qiagen, Hilden, Germany) and quantified using qPCR with the following sets of oligonucleotide primers: *CCND1* promoter, 5′-CGACTGGTCAAGGTAGGAA-3′ (forward) and 5′-CCAAGGGGGTAACCCTAAAA-3′ (reverse).

### Generation of lentiviral plasmid and stable cell lines

The stable cell lines for overexpression were generated by pCDH-CMV-MCS-EF1-copGFP lentiviral vectors (System Biosciences, SBI, Palo Alto, CA, USA) encoding HINT1 WT or HINT1 2KR (K21/30R). The stable cell lines for SIRT1 depletion were generated by pLKO.1 lentiviral vectors encoding shSIRT1 (Sigma-Aldrich, St. Louis, MO, USA). The lentiviral vectors pCDH-HINT1 or pLKO.1 shSIRT1 were cotransfected with packaging vectors into 293T cells using Lipofectamine 3000 (Invitrogen, ThermoFisher Scientific, Carlsbad, CA, USA). After transfection for 24 h, the transfected HEK293T cells were replaced with fresh medium and incubated at 37 °C for 24 h. Then, the supernatant was harvested and filtered using a 0.45-μm syringe filter. DLD1 and SW480 cells were transduced with pCDH-HINT1 WT, pCDH-HINT1 2KR, and pCDH-VEC (negative control) recombinant lentivirus to generate stable overexpression lines. The pCDH stable cell lines were visualized for recombinant plasmid expression using GFP labeling. HEK293T and DLD1 cells were transduced with lentiviral pLK0.1 shSIRT1 and the pLKO.1 shCON (negative control), and then were selected using puromycin (2 μg/ml). Lentivirus was added to cells with 8 μg/ml polybrene (hexadimethrine bromide).

### Cell proliferation assay

Cells were plated at 5 × 10^4^ cells per well in 35-mm plates. The cells were counted every 24 h after transfection. Ten microliters of cell suspension were measured for counting, which was performed with a hemocytometer.

### Cell viability assay

The MTS ([3-(4,5-dimethylthiazol-2-yl)-5-(3-carboxymethoxyphenyl)-2-(4-sulfophenyl)-2*H*-tetrazolium], Promega, Madison, WI, USA) assay was performed to measure cell viability. DLD1 and SW480 stable cell lines were plated in 96-well plates at 1000 cells per well. Then, 20 μl of MTS solution reagent was added to each well, and each plate was incubated at 37 °C for 1–4 h in a humidified, 5% CO_2_ atmosphere. The plates were examined using a 96-well plate reader at 490 nm.

### Colony-forming assay

DLD1 and SW480 stable cell lines were plated in 6-well plates at 1000 cells per well and held at 37 °C for 10–12 days. The colonies were fixed in 80% methanol and stained with 0.5% crystal violet.

### Mouse xenograft model

The animal treatments in this study were approved by the Institutional Animal Care and Use Committee of Ewha Womans University (ESM 16-053). DLD1 stable cell lines (7 × 10^6^), suspended in 100 μl of phosphate-buffered saline, were injected subcutaneously into the flank regions of male BALB/c nude mice, 4–5 weeks old (SLC, Japan). The tumors were allowed to grow to an average volume of ~100 mm^3^. Tumor size was measured using calipers once every 4 days, and tumor volumes were calculated using the following formula: *V* = 1/2 × (width)2 × length. At the end of observation, the mice were killed, and the tumors were homogenized for western blot analysis.

### Statistical analysis

Values are expressed as the mean ± SEM from at least three independent experiments. Significant differences between groups were analyzed using Student’s *t*-test. Statistical significance was inferred at *P < 0.05 and **P < 0.01.

## Results

### HINT1 is acetylated by CBP at K21 and K30 within the HIT domain

**Fig. 1:**
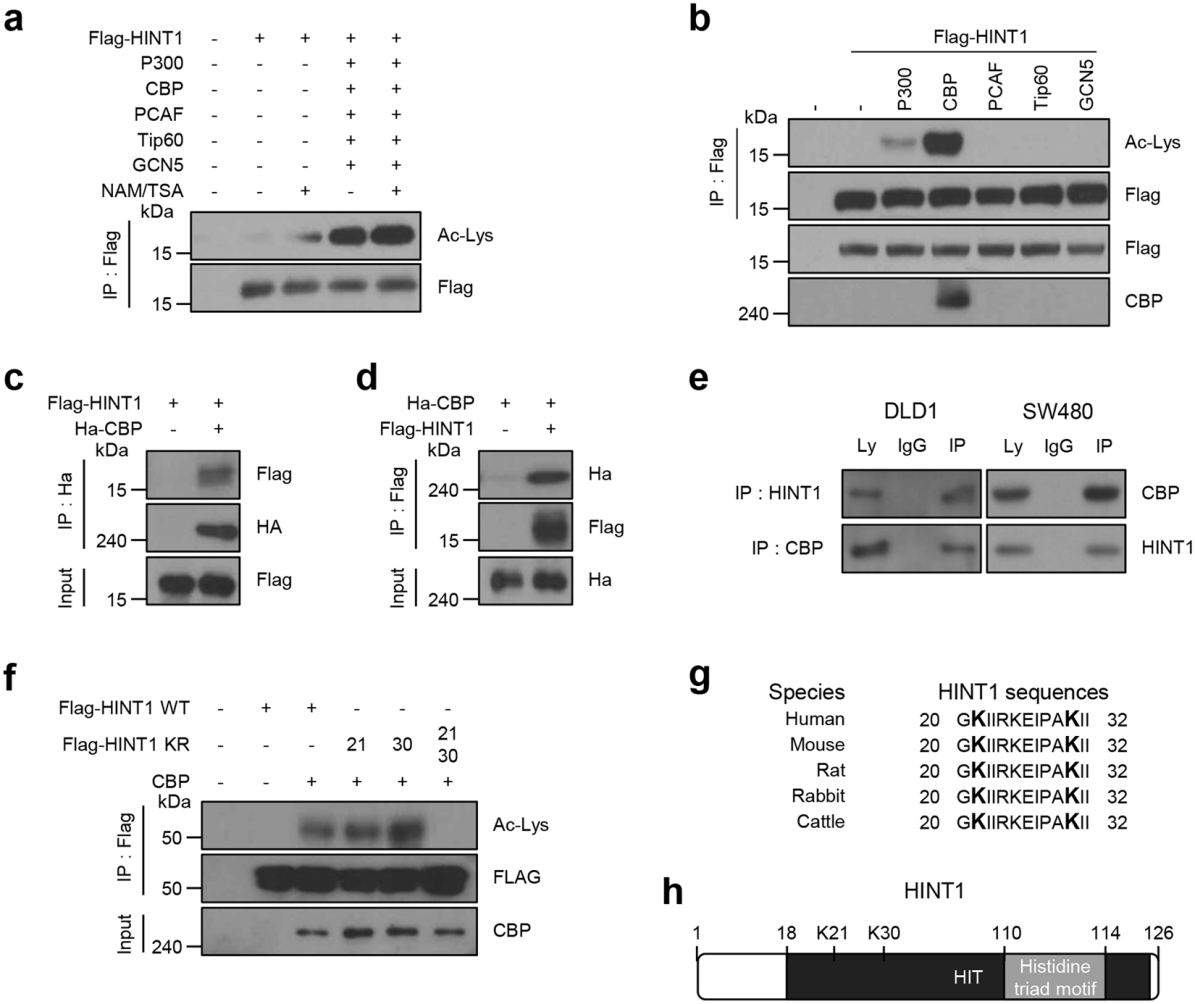
HINT1 binds to and is acetylated by CBP. Flag-HINT1 was coexpressed with all **a** or each **b** of the indicated HATs, p300, CBP, Tip60, pCAF, and Gcn5, in HEK293T cells, and then cells were treated with nicotinamide (NAM, 10 mM for 12 h) and trichostatin A (TSA, 1 µM for 12 h). Acetylation levels of Flag-bead-immunoprecipitated (IPed) HINT1 were determined by immunoblot analysis using pan-acetyl lysine (Ack) antibody. For the reciprocal interaction between HINT1 and CBP, Flag-HINT1 was coexpressed with HA-CBP in HEK293T cells. HINT1 or CBP was immunoprecipitated with HA **c** or Flag **d** antibody, respectively, following western blot analysis using antibodies as indicated. **e** Endogenous HINT1 or CBP proteins in DLD1 or SW480 cells were immunoprecipitated and immunoblotted with antibodies as indicated. **f** CBP was coexpressed with each of the Flag-HINT1 wild-type versions or mutants (K21R, K30R, K21/30R; lysine to arginine) in HEK293T cells. Acetylation levels of Flag-bead-IPed HINT1 were examined by immunoblot analysis. **g** Sequence alignment of the putative acetylation sites K21 and K30 in HINT1 from different species. **h** Schematic representation of the positions of putative acetylation sites K21 and K30 within HINT1.

The PTM database PhosphoSitePLUS (http://www.phosphosite.org) showed that multiple lysine residues within human HINT1, including K21 and K30, are acetylated in various cancer types. A recent study also demonstrated that HINT1 was specifically modified by acetylation at K21, as well as by phosphorylation at S107 and Y109 in activated mast cells^16^. Based on these data, this study sought to identify potential acetyltransferases responsible for HINT1 acetylation. 293T cells were cotransfected with Flag-HINT1 and the major acetyltransferases p300, CBP, pCAF, GCN5, and Tip60, which have been reported to have a critical role in acetylation of most cellular proteins^18^ and were treated with nicotinamide (NAM), an inhibitor of the sirtuin family, and trichostatin A (TSA), an inhibitor of histone deacetylase (HDAC) I/II. Western blotting with a pan anti-acetyl lysine antibody (Ac-K) showed that HINT1 was indeed modified by acetylation (Fig. 1a). Among the acetyltransferases analyzed, CBP significantly elevated the acetylation of HINT1, but not of the others (Fig. 1b). In support of this result, reciprocal interactions were also found between overexpressed Flag-HINT1 and HA-CBP in 293T cells (Fig. 1c, d), as well as between endogenous HINT1 and CBP in DLD1 and SW480 cells (Fig. 1e). These data suggest that CBP is a major acetyltransferase of HINT1.

Analysis of the PTM database PhosphoSitePLUS revealed that there are two putative acetylated lysines (K21 and K30) that are highly conserved in diverse species and commonly detected in several types of cancer cells. Using this as background, to determine which lysine residue(s) are acetylated by CBP, we replaced one or both of the two acetylated lysine residues (K21 and K30) in HINT1 with arginine (R), an amino acid residue mimicking the deacetylation form, and investigated the acetylation status of those mutants using western blot analysis with primary Ac-K antibody. Acetylation was still detected in each one-lysine mutant, HINT1 K21R or K30R alone, but not in the two-lysine mutant, HINT1 2KR (K21/30R) (Fig. 1f), indicating that both K21 and K30 within HINT1 are modified via acetylation by CBP. Moreover, both lysine residues are positioned within the HIT domain of the HIT superfamily and are well conserved in diverse mammalian organisms (Fig. 1g, h), suggesting that both might be implicated in HINT1 function.

### HINT1 is deacetylated by SIRT1

**Fig. 2:**
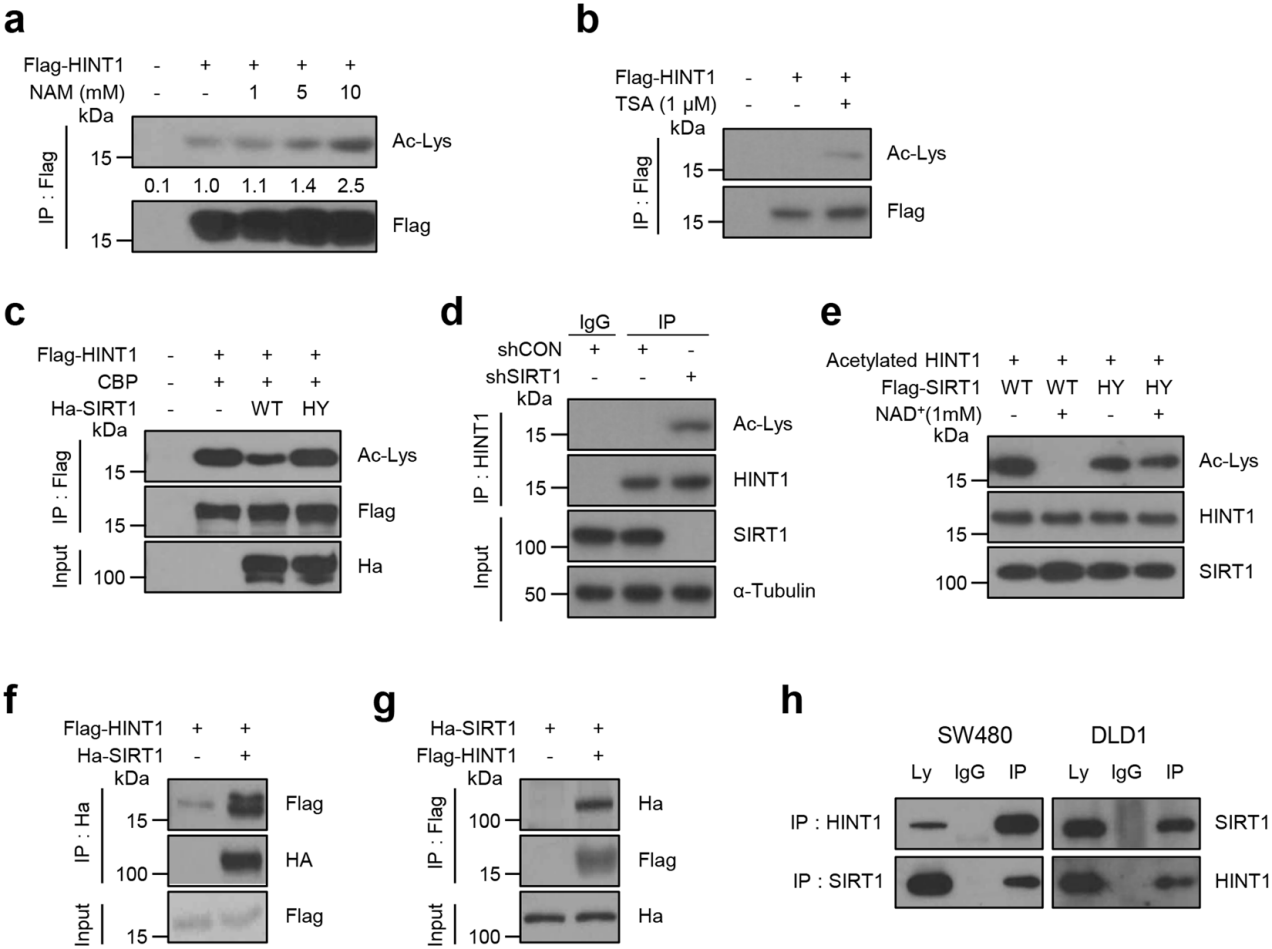
HINT1 binds to and is deacetylated by SIRT1. **a**, **b** Flag-HINT1 was overexpressed in HEK293T cells, and then the cells were treated with **a** NAM for 24 h or **b** TSA for 6 h at different concentrations as indicated. **c** Flag-HINT1 was coexpressed with CBP, wild-type SIRT1 (WT), or SIRT1-catalytic mutant (H363Y) in HEK293T cells. **a**–**c** The acetylation levels of Flag-bead-IPed HINT1 were determined by western blot analysis. **d** SIRT1 was knocked down by a lentiviral system (shSIRT1) in DLD1 cells. Acetylation levels of HINT1 IPed with its antibody were determined by western blot assay. **e** For the in vitro deacetylation assay, 1 μg of the acetylated HINT1 protein was incubated with recombinant SIRT1 WT or SIRT1 HY in the presence or absence of NAD^+^ (1 mM) at 37 °C for 2 h, followed by western blot analysis. **f**, **g** For the reciprocal interaction between HINT1 and SIRT1, Flag-HINT1 was coexpressed with HA-SIRT1 in HEK293T cells. SIRT1 or HINT1 was immunoprecipitated with HA (**f**) or Flag (**g**) antibody, respectively, following western blot analysis using antibodies as indicated. **h** Endogenous HINT1 or SIRT1 proteins in SW480 or DLD1 cells were immunoprecipitated and immunoblotted with antibodies as indicated.

A previous proteomics study revealed that the acetylation level of HINT1 protein was enhanced by treatment with NAM, an inhibitor of pan-sirtuin inhibitor, and by SIRT1 deletion in HeLa cells^27^, indicating that HINT1 may be a potential target of the sirtuin family. Thus, this study examined first whether the sirtuin family is involved in the deacetylation of HINT1. For this experiment, the acetylation level of HINT1 was examined after treatment with several doses of NAM in 293T cells. Consistent with a previous report, nicotinamide elevated the acetylation level of HINT1 in a dose-dependent manner (Fig. 2a). We also determined whether classic HDACs are involved in the deacetylation of HINT1. Interestingly, the acetylation level of HINT1 was also increased by TSA, a known class I and II HDAC inhibitor (Fig. 2b), indicating that deacetylation of HINT1 is regulated by both the sirtuin family and classic HDACs.

Next, to determine whether SIRT1 is a potential deacetylase of HINT1, in vitro and in vivo deacetylation analyses were performed. After Flag-tagged HINT1 was coexpressed with wild-type SIRT1 (SIRT1 WT) or SIRT1-catalytic inactive mutant (SIRT1 H363Y) in 293T cells, in vivo deacetylation analysis was employed using a western blot assay. The results showed that the expression of SIRT1 WT, but not SIRT1 HY (H363Y), led to a significant decrease in the in vivo HINT1 acetylation level (Fig. 2c), indicating that SIRT1 acts as a potential deacetylase for HINT1. Consistently, the knockdown of SIRT1 also led to an increased acetylation level in endogenous HINT1 protein (Fig. 2d). Next, to exclude the possibility that other sirtuin members (SIRT2–7) might be implicated in the deacetylation of HINT1, in vitro deacetylation analysis was performed. The acetylated HINT1 protein was purified by immunoprecipitation using Flag-agarose in 293T cells, which were coexpressed with Flag-HINT1 and CBP and then incubated with recombinant SIRT1 WT or SIRT1 HY in vitro, followed by western blot assay with Ac-K antibody. When SIRT1 WT, but not SIRT1 HY, was incubated with the acetylated form of HINT1, a significant decrease in HINT1 acetylation was observed in an NAD^+^-dependent direction (Fig. 2e), connecting HINT1 deacetylation directly to SIRT1-catalytic activity. Consistently, in an attempt to analyze HINT1–SIRT1 interactions, the study also showed reciprocal interactions between overexpressed Flag-HINT1 and HA-SIRT1 in 293T cells (Fig. 2f, g), as well as between endogenous HINT1 and SIRT1 in DLD1 and SW480 cells (Fig. 2h). Taken together, these results support that SIRT1 binds to and deacetylates HINT1.

### SIRT1 deacetylates HINT1 to inhibit the transcriptional activity of β-catenin

**Fig. 3:**
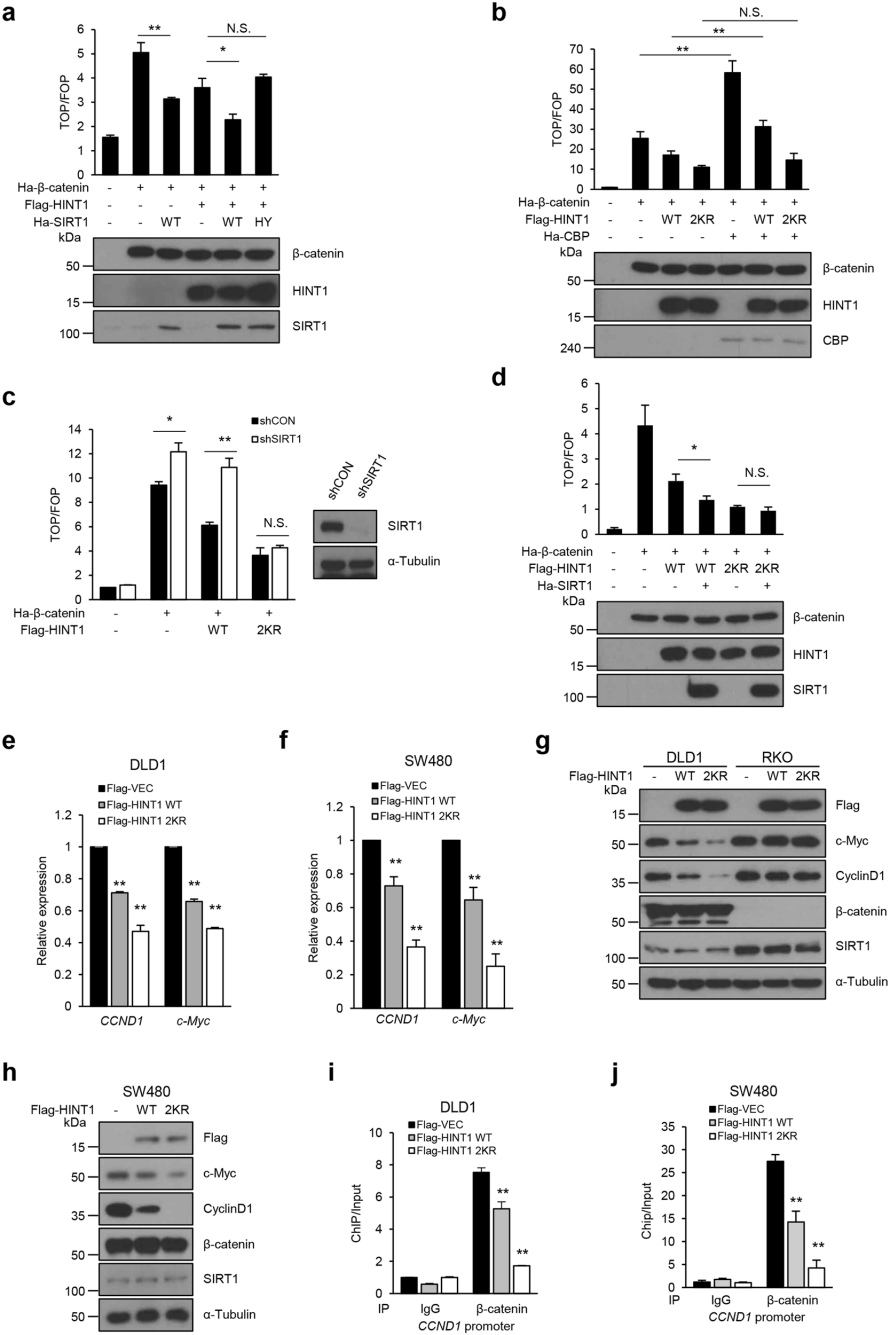
HINT1 deacetylation by SIRT1 attenuates the transcriptional activity of β-catenin. Luciferase reporter plasmids containing the TCF/LEF binding sequence (TOP/FLASH) were cotransfected into HEK293T cells with several vectors, as indicated. The relative transcriptional activity was determined by the dual luciferase reporter assay. The FOP/FLASH construct was used as a negative control. **a** Cotransfection with HA-β-catenin, Flag-HINT1, HA-SIRT1 WT, or HY. **b** Cotransfection with HA-β-catenin, HA-CBP, Flag-HINT1 WT, or 2KR. **c** HA-β-catenin and Flag-HINT1 WT or 2KR were coexpressed in control (shCON) or SIRT1 (shSIRT1) knockdown cells. **d** Cotransfection with HA-β-catenin, HA-SIRT1 WT, Flag-HINT1 WT or 2KR. **e**–**j** Flag-HINT1 WT or 2KR was transfected into DLD1, RKO, or SW480 cells, followed by quantitative real-time PCR (qRT-PCR) to determine the mRNA level of cyclin D1 and c-Myc genes in DLD1 (**e**) or SW480 cells (**f**). Western blot analysis to determine the protein levels of cyclin D1 and c-Myc gene in DLD1 (**g**), RKO (**g**), or SW480 cells (**h**), and chromatin IP (ChIP) analysis to determine the recruitment levels of β-catenin on the promoter of the CCND1 gene in DLD1 (**i**) or SW480 cells (**j**). All data are the mean ± SEM of three independent experiments. *P < 0.05, **P < 0.01.

Because previous studies have shown that HINT1 acts as a tumor suppressor by inhibiting the transcriptional activities of several oncogenic transcription factors, such as β-catenin and MITF, in various cancer cell types^8,11^, this study evaluated whether reversible acetylation of HINT1 might affect the transcriptional activity of β-catenin in colon cancer cells. To investigate the effects of HINT1 acetylation or deacetylation on the transcriptional activity of β-catenin, reporter constructs containing the TCF/LEF binding sites (TOP/FLASH) as well as their negative control (FOP/FLASH) were used in DLD1 cells^8^. Consistent with previous reports, transfection of the HINT1 WT construct into cells reduced the transcriptional activity of β-catenin (Fig. 3a). Next, the study analyzed whether SIRT1 could affect the transcriptional activity of β-catenin through HINT1. Interestingly, although expression of SIRT1 WT significantly suppressed the relative transcriptional activity of β-catenin compared to expression in the mock control or HINT1 WT alone, SIRT1 HY, a catalytic inactive mutant, did not (Fig. 3a). In contrast, overexpression of CBP, which might be a potential acetyltransferase of HINT1, markedly enhanced the transcriptional activity of β-catenin compared to that with mock control or HINT1 WT expression alone (Fig. 3b). However, CBP-mediated increase of β-catenin activity was not observed with the expression of HINT1 2KR, a deacetylation mimetic mutant (Fig. 3b). These results suggest that HINT1 deacetylation promotes the transcriptional activity of β-catenin, whereas HINT1 acetylation activates it. Consistent with this notion, knockdown of SIRT1 (shSIRT1) resulted in increased transcriptional activity of β-catenin compared to that in the control (shCON) group (Fig. 3c). Furthermore, while knockdown of SIRT1 in cells overexpressing HINT1 WT led to notably enhanced activity of β-catenin, this did not take place in cells overexpressing HINT1 2KR (Fig. 3c). Similar to these results, overexpression of HINT1 2KR led to marked inhibition of β-catenin activity regardless of overexpression of SIRT1 WT (Fig. 3d). These results suggest that reversible acetylation of HINT1 at K21 and 30 by CBP and SIRT1 can modulate the transcriptional activity of β-catenin.

To further validate the results obtained from the reporter system, after overexpression of HINT1 WT or 2KR in DLD1 and SW480 cells, the endogenous expression levels of β-catenin target genes such as cyclin D1 and c-myc were also examined by real-time RT-PCR and western blot assay. Consistently, the mRNA and protein levels of cyclin D1 and c-myc were significantly decreased in cells overexpressing the HINT1 2KR mutant compared to those overexpressing HINT1 WT (Fig. 3e–h). More importantly, unlike DLD1 cells whose β-catenin is constitutively active, RKO cells whose β-catenin is inactive, did not display the altered expression levels of cyclin D1 and c-myc with overexpression of HINT1 WT or 2KR (Fig. 3g).

Interestingly, it was also found that the recruitment of β-catenin to the promoter of the *CCND1* gene was more markedly decreased in DLD1 and SW480 cells transfected with the HINT1 2KR construct than in those transfected with the HINT1 WT construct (Fig. 3i, j), indicating that HINT1 may bind to and suppress the ability of β-catenin to access the promoter of its target genes, which can be regulated by reversible acetylation of HINT1. Taken together, these results further confirm that reversible acetylation could act as a major mechanism for the regulation of HINT1-mediated inhibition of β-catenin.

### Reversible acetylation by CBP and SIRT1 regulates the binding capacity of HINT1 for β-catenin

**Fig. 4:**
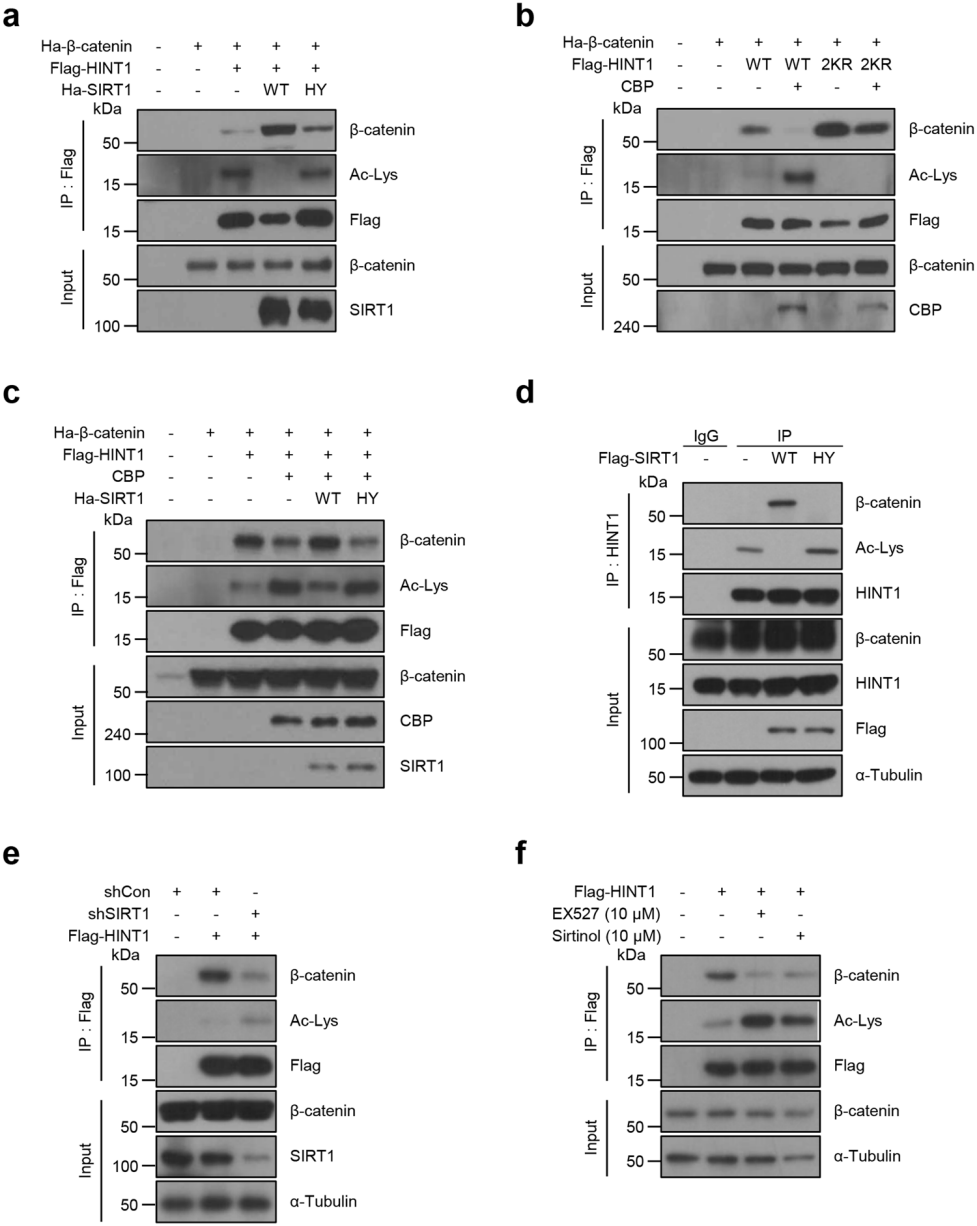
Reversible acetylation of HINT1 by CBP and SIRT1 regulates the binding capacity of HINT1 for β-catenin. **a**–**c** HEK293T cells were cotransfected with several expression vectors and then subjected to IP and western blot assays using antibodies as indicated. **a** Coexpression of HA-β-catenin, Flag-HINT1, and HA-SIRT1 WT or HY. **b** Coexpression of HA-β-catenin, CBP, and Flag-HINT1 WT or 2KR. **c** Coexpression of HA-β-catenin, Flag-HINT1, CBP, and HA-SIRT1 WT or HY. **d** Flag-SIRT1 WT or HY was expressed in DLD1 cells, and IP and western blot assays were performed using antibodies as indicated. **e** DLD1 cells in which SIRT1 was knocked down by a lentiviral system (shSIRT1) were transfected with Flag-HINT1. **f** DLD1 cells were transiently transfected with Flag-HINT1 and treated with EX527 (10 µM) and sirtinol (10 µM) for 12 h. **e**, **f** Followed by IP and western blot assays using antibodies as indicated.

Previous studies showed that the interaction of HINT1 with MITF can be disrupted by PTMs, including acetylation or phosphorylation and Ap4A; this interaction occurs via a side reaction by LysRS (Lysyl-tRNA synthetase)^15,16^. Thus, we also investigated the possibility that reversible acetylation by CBP and SIRT1 could regulate the HINT1-mediated inhibition of β-catenin activity through mediating the capacity of HINT1 to bind to β-catenin. After the Flag-HINT1 WT or 2KR construct was cotransfected with HA-SIRT1 WT, HY, or CBP into 293T cells, the acetylation status and capacity of HINT1 to bind to β-catenin were examined through immunoprecipitation and western blot assays. Overexpression of SIRT1 WT, but not SIRT1 HY, resulted in an elevated capacity of HINT1 to bind to β-catenin, as well as reduced HINT1 acetylation (Fig. 4a), suggesting that deacetylation by SIRT1 increases the ability of HINT1 to bind to β-catenin. Consistently, overexpression of CBP led to a decreased capacity of HINT1 WT to bind to β-catenin, as well as increased acetylation of HINT1, but did not have these effects on HINT1 2KR (Fig. 4b). Moreover, conversely, the effect of CBP on the capacity of HINT1 to bind to β-catenin was reversed by the expression of SIRT1 WT but not by SIRT1 HY (Fig. 4c). More importantly, expression of SIRT1 WT, but not SIRT1 HY, led to diminished ability of the endogenous HINT1 protein to bind to β-catenin, as well as to reduced acetylation of this protein (Fig. 4d). To further corroborate this finding, we examined the acetylation levels and function of HINT1 under SIRT1 knockdown or treatment with SIRT1 inhibitors. Loss of SIRT1 in DLD1 cells led to a significant increase in the acetylation level of HINT1 and a decrease in the binding capacity of HINT1 with β-catenin compared to that in the shCon cells (Fig. 4e). Consistent with this result, treatment with SIRT1 inhibitor EX527 or sirtinol also augmented the acetylation level of HINT1 and markedly decreased its binding capacity for β-catenin (Fig. 4f). Overall, these findings indicate that reversible acetylation by CBP and SIRT1 has an important role in modulating the association of HINT1 with β-catenin.

### Deacetylation of HINT1 promotes its tumor-suppressive function in colon cancer cell lines and in an in vivo xenograft mouse model

**Fig. 5:**
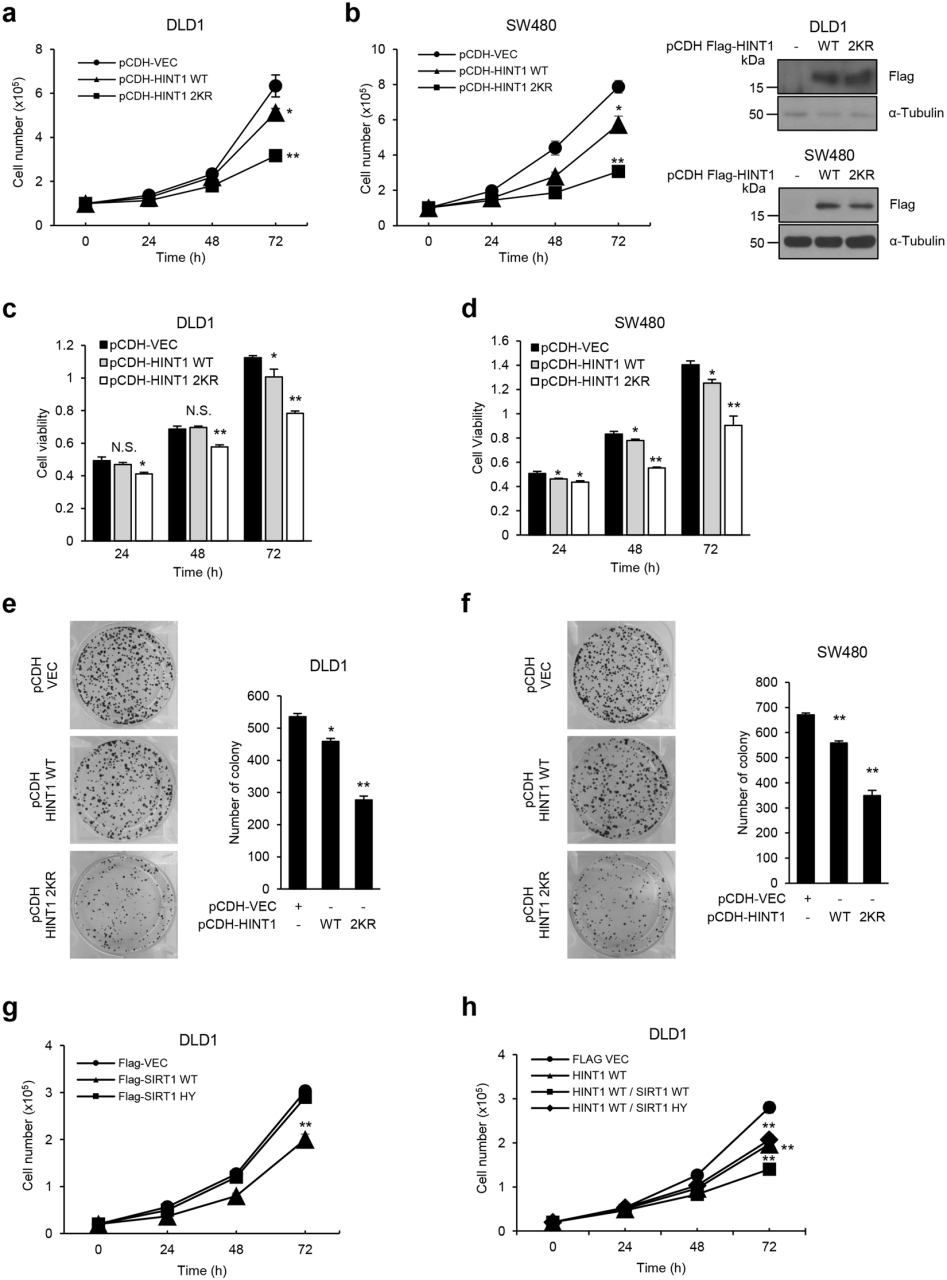
SIRT1-mediated deacetylation of HINT1 promotes its tumor-suppressive function in colon cancer cell lines. DLD1 and SW480 cells were infected with control or Flag-HINT1 WT or 2KR-expressing lentivirus for 24 h. Lentiviral-infected DLD1 (**a**, **c**) and SW480 cells (**b**, **d**) were grown in a 96-well culture plate, and then cell counting was performed (**a**, **b**) and MTS assay (**c**, **d**) at the indicated times to determine cell proliferation rates. The same lentiviral-infected cell lines were grown for 14 days. The colonies formed by DLD1 (**e**) and SW480 cells (**f**) were stained and counted. Representative images are shown. Each Flag-SIRT1 WT or HY-expressing construct was transfected alone (**g**) or cotransfected (**h**) with the HINT1 WT construct into DLD1 cells, and then cell counting was performed to determine cell proliferation rates. All data are the mean ± SEM of three independent experiments. *P < 0.05, **P < 0.01.

As HINT1 has been previously described as a tumor suppressor in several cancer cells and as an inhibitor of β-catenin activity in colon cancer and melanoma^2^, this study examined the physiological roles of the constitutive deacetylated HINT1 mutant (HINT1 2KR) in tumorigenesis in DLD1 and SW480 cell lines. This study assessed the ability of colon cancer cells, which were stably transfected with the HINT1 WT or 2KR construct, to proliferate, form colonies, and survive. Stable expression of the HINT1 2KR mutant led to a much slower rate of proliferation in both cell lines compared to that with HINT1 WT expression (Fig. 5a, b). Likewise, overexpression of the HINT1 2KR mutant also significantly diminished cell viability (Fig. 5c, d) and the number of colonies formed (Fig. 5e, f) when compared with the results for HINT1 WT, indicating that HINT1 deacetylation promotes its tumor-suppressive function in the colon cancer cell lines DLD1 and SW480. To further validate this notion, the study next investigated whether deacetylation of HINT1 by SIRT1 also promotes the HINT1 tumor-suppressive function. Because SIRT1 was previously reported to act as either a tumor suppressor or promoter, depending on the cancer cell type and context of cells, this study first assessed the role of SIRT1 in the tumorigenesis of DLD1 cells. Similar to the results of some previous studies^23,24^, the results showed that ectopic expression of SIRT1 WT, but not SIRT1 HY, displayed reduced cell growth (Fig. 5g). Furthermore, overexpression of SIRT1 WT in HINT1 WT-expressing stable cells also decreased cell proliferation, but this effect did not take place with SIRT1 HY (Fig. 5h). In contrast, treatment of DLD1 cells expressing HINT1 WT with SIRT1 inhibitor EX527 or sirtinol led to an increased cell proliferation rate, but this effect of SIRT1 inhibitors did not occur in DLD1 cells expressing the HINT1 2KR mutant (Supplementary Fig. S1a, b). These results suggest that HINT1 deacetylation by SIRT1 stimulates its tumor-suppressive activity in colon cancer cell lines.

**Fig. 6:**
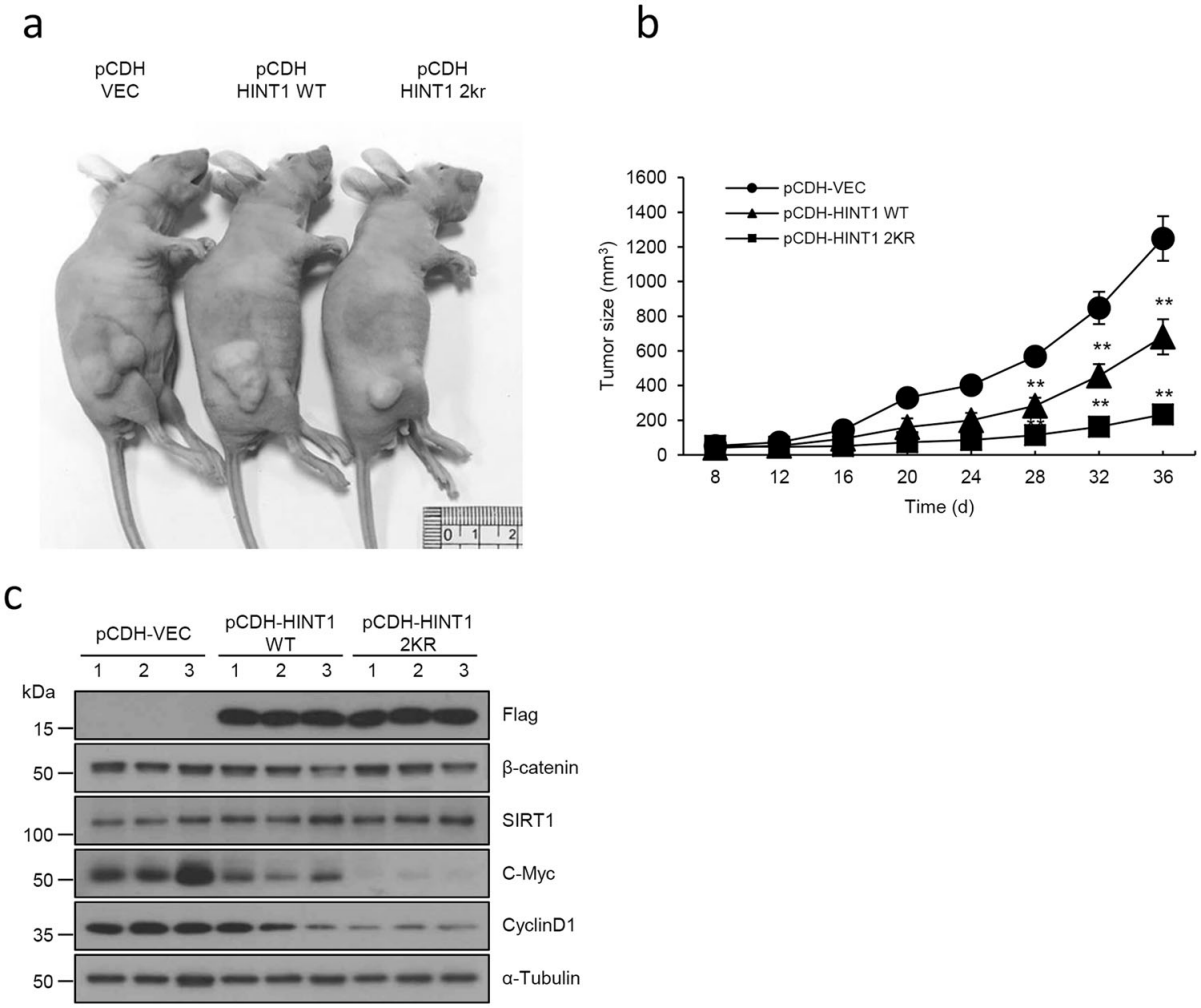
The deacetylated HINT1 mutant (HINT1 2KR) suppresses tumorigenesis in an in vivo xenograft mouse model. Nude mice (five mice/group) were injected with 1 × 10^7^ DLD1 cells stably transfected with mock control or with the Flag-HINT1 WT or 2KR construct. The xenograft tumors were measured at the indicated times and dissected at the endpoint. **a** Representative images of mice are shown. Scale bar: 1 cm. **b** Quantification of the average volume of tumors as indicated times is shown. The data show the mean ± SEM versus negative control pCDH-VEC. **P < 0.01. **c** Protein levels of cyclin D1 and c-Myc were determined by western blot assay in tumors derived from three nude mice from each group.

To further confirm this notion in vivo, we examined in vivo tumorigenesis by injecting DLD1 cells stably expressing HINT1 WT or HINT1 2KR into nude mice. Indeed, stable expression of the HINT1 2KR mutant led to a much smaller tumor size than the control or HINT1 WT (Fig. 6a, b). Consistent with this result, stable expression of the HINT1 2KR mutant, a constitutive deacetylation mimetic mutant, led to significantly decreased expression of β-catenin target genes such as cyclin D1 and c-myc (Fig. 6c). Taken together, these results indicate that reversible acetylation of HINT1 by CBP and SIRT1 may be a major mechanism for regulation of HINT1 tumor-suppressive activity.

### HINT1 deacetylation also promotes the capacity of HINT1 to bind to MITF to inhibit cell growth in the A375 melanoma cell line

**Fig. 7:**
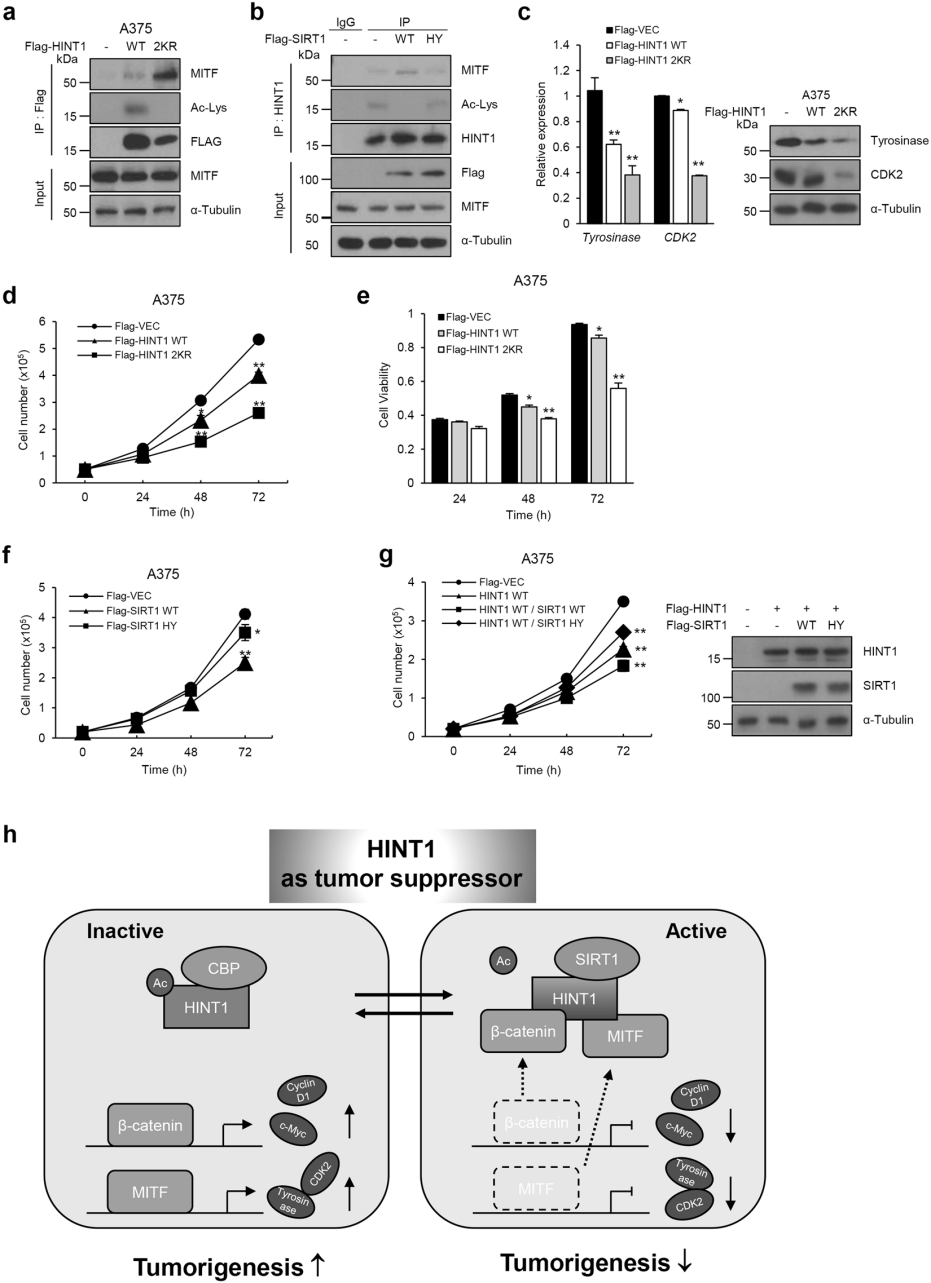
Deacetylation of HINT1 by SIRT1 also promotes its inhibition of MITF to suppress cell growth in A375 cell lines. A375 cells were transfected with Flag-HINT1 WT or 2KR mutant and subjected to IP and western blot assays using antibodies as indicated. **b** A375 cells were transfected with the Flag-SIRT1 WT or HY mutant and subjected to IP and western blot assays using antibodies as indicated. **c** A375 cells were transfected with Flag-HINT1 WT or 2KR vectors and subjected to qRT-PCR, and western blot assays to determine the mRNA and protein levels of tyrosinase and Cdk2. **d**, **e** A375 cells were transfected with Flag-HINT1 WT or 2KR vectors followed by cell counting (**d**) and MTS assay (**e**) at the indicated times. **f** Flag-SIRT1 WT or HY was expressed in A375 cells, and then cell counting was performed to determine cell proliferation rates. **g** Flag-SIRT1 WT or HY constructs were cotransfected with the HINT1 WT construct in A375 cells, and then cell counting was performed to determine cell proliferation rates, and western blotting was performed to confirm the expression levels of expression vectors as indicated. **h** Proposed model depicting how reversible acetylation of HINT1 by SIRT1 and CBP regulates its tumor-suppressive activity in cancer cells. All data are the mean ± SEM of three independent experiments. *P < 0.05, **P < 0.01.

Similar to β-catenin, previous studies demonstrated that HINT1 could also interact with and repress the function of MITF, which has been well known as a master regulator of melanocyte development and function, in activated mast and melanoma cells^8,11,15^. Thus, we further explored whether reversible acetylation of HINT1 could affect the interaction of HINT1 with MITF and then affect MITF-mediated oncogenic function in the A375 melanoma cell line. After transfection of the HINT1 WT or 2KR construct into the A375 colon cancer cell line, the association of HINT1 with MITF and the endogenous expression levels of MITF target genes, including tyrosinase and CDK2, were investigated. Ectopic expression of HINT1 2KR led to significantly increased interaction between endogenous HINT1 and MITF compared to that in HINT1 WT-expressing cells (Fig. 7a). Consistently, expression of SIRT1 WT in A375 cells also caused elevated binding of endogenous HINT1 to MITF, as well as a marked reduction in acetylation levels, compared to that in cells expressing SIRT1 HY (Fig. 7b). Indeed, qRT-PCR and western blot analysis revealed that expression of the HINT1 2KR mutant led to marked upregulation of MITF target genes, including tyrosinase and CDK2, at the mRNA and protein levels compared to that with expression of the HINT1 WT construct (Fig. 7c), further validating the effect of reversible acetylation on the ability of HINT1 to inhibit MITF.

As HINT1 was previously reported to act as a tumor suppressor in melanoma cells^2^, this study next evaluated whether deacetylation of HINT1 by SIRT1 also activates its tumor-suppressive activity. The A375 colon cancer cells were stably transfected with lentiviral vectors expressing either HINT1 WT or HINT1 2KR, and then the cell proliferation rate and cell viability were examined. As shown in Fig. 7d, e, the HINT1 2KR-expressing A375 cell lines grew more slowly than the HINT1 WT-expressing stable A375 cell lines. To further determine whether deacetylation by SIRT1 affects the tumor-suppressive activity of HINT1, A375 cells were transfected with only SIRT1 WT or HY, and then the cell proliferation rates were examined. Although the role of SIRT1 in tumorigenesis in melanoma cells has been controversial, our data showed that overexpression of SIRT1 WT, but not SIRT1 HY, led to a reduced cell proliferation and cell viability rate compared to that of the mock control (Fig. 7f; Supplementary Fig. S2a). Furthermore, coexpression of HINT1 WT with SIRT1 WT, but not SIRT1 HY, also decreased the cell growth and cell viability rates compared to those with HINT1 WT expression alone (Fig. 7g; Supplementary Fig. S2b). These results suggest that deacetylation by SIRT1 also promotes HINT1-mediated inhibition of MITF to inhibit tumorigenesis in A375 cells.

## Discussion

There is accumulating evidence that HINT1 might be a novel tumor suppressor, with action unrelated to its enzymatic activity as a nucleotide hydrolase and/or its transferase activity^1,2^. The function of HINT1 as a tumor suppressor is supported by observations that a deficiency of HINT1 in mice led to increased susceptibility to both spontaneous and carcinogen-induced tumor formation^4,5^. Interestingly, several previous studies have revealed that HINT1 can bind to several transcriptional factors, including β-catenin, MITF, and AP1, suppress their oncogenic transcriptional activities, and thus inhibit tumorigenic properties in several cancer cell types^8,11,15^. However, currently, little clinical relevance of HINT1 expression or mutation in several human cancers has been reported^28–30^. This suggests the possibility that dysfunction of HINT1-mediated tumor-suppressive activity can be governed by mechanisms other than its expression level in cancers.

Recent studies indicate that the transcriptional activity of MITF, which has been well reported as a master regulator of melanocyte development and tumorigenesis, is inhibited by HINT1 through direct binding^2^. This association between MITF and HINT1 is disrupted by the binding to HINT1 of the second messenger Ap4A, which is mainly produced by lysyl-tRNA synthetase^15^. More importantly, the most recent study showed that HINT1 was subjected to K21 acetylation and Y109 phosphorylation in activated mast cells^16^, and these modifications promote the formation of colonies of melanoma cells derived from human melanoma patients in soft agar, indicating a PTM-dependent regulatory mechanism that governs the binding capacity of HINT1 for MITF in melanoma cells^16^. However, the detailed mechanisms are not yet understood. This study provides a detailed regulatory mechanism and shows the effect of HINT1 acetylation on tumorigenesis. The present data reveal that CBP- and SIRT1-driven reversible acetylation of HINT1 at both K21 and K30 affects the capacity of HINT1 to bind to β-catenin or MITF, which, in turn, can influence its tumor-suppressive activity in cancer cell lines and in an in vivo xenograft mouse model.

Although a recent study identified the acetylation at K21 of HINT1 using mass spectrometry analysis and database searches^16^, the present study identified the effect at K30 as well as K21 using a western blot assay with a pan-acetyl lysine antibody. A recent study revealed that ectopic expression of the acetylation mimetic HINT1 mutant (HINT1 K21Q; lysine to glutamine or K21D; lysine to aspartate) promoted colony formation in soft agar but did not promote cell proliferation compared to that with wild-type HINT1 expression in cancer cells derived from melanoma patients^16^, indicating that acetylation of HINT1 might affect cell migration. Unlike in a previous report^16^, the present study showed that the deacetylation mimetic HINT1 mutant (at both K21R and K30R) inhibits cell proliferation in DLD1 colon cancer cell lines and A375 melanoma cell lines, although the study did not include a soft agar assay; the findings indicate that reversible acetylation of HINT1 at both K21 and K30 might have an important role in cell proliferation. In support of this, the study also demonstrated that ectopic expression of HINT1 2KR markedly promoted the association of HINT with MITF, which then led to a significant decrease in the expression of tyrosinase and Cdk2, which has been reported to be a target of MITF. Consistently, overexpression of the deacetylated HINT1 mutant (HINT1 2KR) also augmented the capacity of HINT1 to bind to β-catenin, repressed the expression of its target genes cyclin D1 and c-Myc, and resulted in notably decreased tumorigenesis. Given these results, this study demonstrates that reversible acetylation of HINT1 at both K21 and K30 may have a key role in its tumor-suppressive function by modulating the association of HINT1 with β-catenin or MITF.

In the past decade, protein lysine acetylation, which takes place reversibly by way of HATs and HDACs, including the sirtuin family, has been reported to be involved in various human diseases, including cancer^18,19^. This study identifies CBP and SIRT1 as major acetyltransferase and deacetylases of HINT1, respectively. Dysregulation of the transcriptional and epigenetic functions of CBP is associated with leukemia and other types of cancer, as has been reported in numerous previous studies; thus, CBP has been recognized as a potential target for anticancer drugs. In colon cancer cells, although CBP associates with β-catenin and activates the set of its target genes linked to cell proliferation in an acetylation-dependent manner, the role of CBP in tumorigenesis is still controversial, perhaps due to a dual role for CBP in both enhancing and suppressing Wnt signaling in a context-dependent fashion^31,32^. This study shows that HINT1 is a novel potential target of CBP. In addition, the analysis also shows that acetylation of HINT1 by CBP promotes the release of β-catenin from the HINT1-β-catenin complex to enhance the transcriptional activity of β-catenin, which in turn boosts oncogenic features in cancer. Thus, the results suggest that the role of CBP, which promotes tumorigenesis by activating Wnt signaling in cancer, may be accomplished by upregulating HINT1 and β-catenin acetylation.

In addition, numerous studies implicate SIRT1 in tumorigenesis in various cancer types, including colon cancer and melanoma cells, but its function in most cancer cells is still under debate^20–22^ because of its controversial roles in tumorigenesis, which are dependent on cell type or context. In colon or melanoma cancer cells, SIRT1 binds to and deacetylates several transcriptional regulators, including β-catenin and p53, which modulates tumorigenic properties such as cell proliferation and apoptosis^20–22^. In support of previous proteomic study^27^, present study also shows that HINT1 is a new target of SIRT1. Moreover, HINT1 deacetylation by SIRT1 promotes the association of HINT1 with β-catenin, thus inhibiting carcinogenesis through downregulating β-catenin oncogenic activity, which suggests that SIRT1 might serve as a tumor suppressor through deacetylation of HINT1 and β-catenin in colon cancer cells. In fact, overexpression of HINT1 2KR, a deacetylation mimetic HINT1 mutant, caused a significant decrease in tumor formation, further confirming that reversible acetylation of HINT1 might have an important role in HINT1-mediated tumor-suppressive activity. Taken together, the results of this study indicate that the tumor-suppressive function of HINT1 can be regulated by an acetylation-dependent mechanism involving CBP and SIRT1. In addition, these results also suggest that CBP and SIRT1 can regulate Wnt signaling and ultimately tumorigenesis by reversible acetylation of HINT1 and β-catenin.

In conclusion, this study demonstrates that reversible acetylation of HINT1 at K21 and K30 by CBP and SIRT1 modulates the capacity of HINT1 to bind to β-catenin or MITF and in turn to control tumorigenic features in colon cancer and melanoma cells (Fig. 7h). Although studies are currently focusing on the clinical relevance of HINT1 expression in several human cancers, the results here suggest that HINT1 modification by PTMs, such as phosphorylation and acetylation, should be assessed to determine their impact on the clinical implication of HINT1 in human cancers.

## Supplementary information

Supplementary Information
