# Supplementary material for: Deacetylation by SIRT1 promotes the tumor-suppressive activity of HINT1 by enhancing its binding capacity for β-catenin or MITF in colon cancer and melanoma cells
Source: Exp Mol Med. 2020 Jul 7;52(7):1075–89. doi: 10.1038/s12276-020-0465-2 (PMC8080686; doi:10.1038/s12276-020-0465-2)
Supplement: Supplementary file 1 — Supplementary Information [file 12276_2020_465_MOESM1_ESM.pdf]

Supplementary Figure S1. SIRT1 inhibitor EX527 and sirtinol decreased the HINT1 WT -mediated tumor suppressive activity but not HINT1 2KR.

(a-b) DLD1 cells were overexpressed with HINT1 WT or 2KR mutant and treated with or without EX527 (a) or sirtinol (b), followed by cell counting at the indicated times. All data are the mean ± SEM of three independent experiments. *$P < 0.05$, **$P < 0.01$.

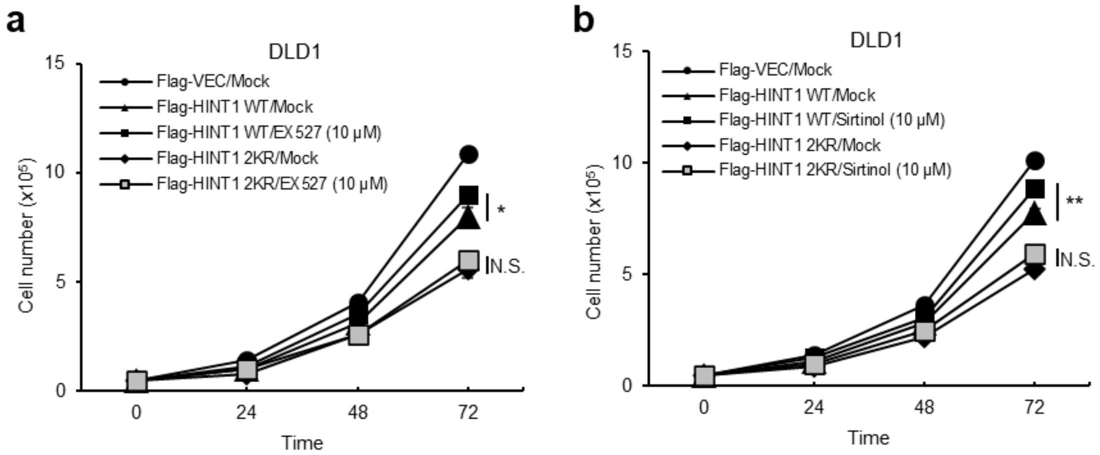

Jung et al., Sup Fig1

Supplementary Figure S2. SIRT1 and HINT1 act synergistically to inhibit viability in A375 cells.

(a-b) MTS assay was performed at the indicated times to determine viability in A375 cells which were transfected with (a) SIRT1 WT or HY and co-transfected with (b) HINT1 WT and SIRT1 WT or HY. All data are the mean ± SEM of three independent experiments. *$P < 0.05$, **$P < 0.01$.

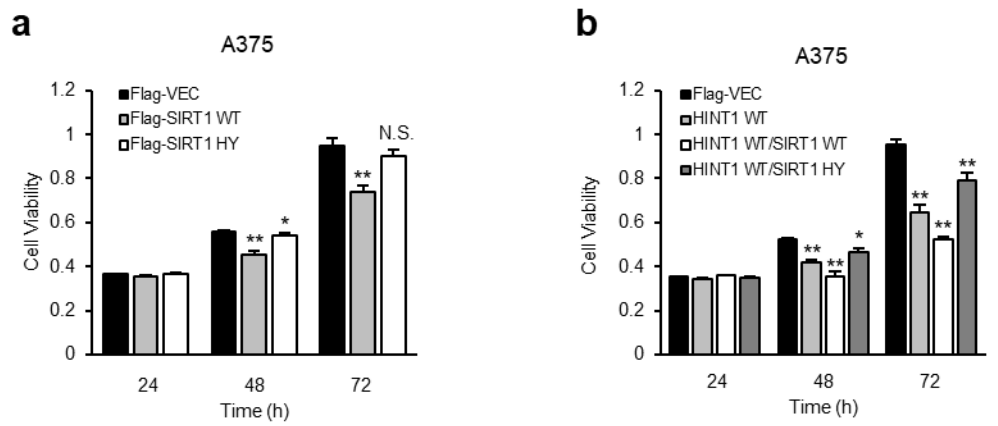

Jung et al., Sup Fig2
